# Combined obeticholic acid and elafibranor treatment promotes additive liver histological improvements in a diet-induced *ob/ob* mouse model of biopsy-confirmed NASH

**DOI:** 10.1038/s41598-019-45178-z

**Published:** 2019-06-21

**Authors:** Jonathan D. Roth, Sanne S. Veidal, Louise K. D. Fensholdt, Kristoffer T. G. Rigbolt, Romeo Papazyan, Jens Christian Nielsen, Michael Feigh, Niels Vrang, Mark Young, Jacob Jelsing, Luciano Adorini, Henrik H. Hansen

**Affiliations:** 1 Intercept Pharmaceuticals, San Diego, CA USA; 2 Gubra, Hørsholm, Denmark

**Keywords:** Gastroenterology, Drug discovery

## Abstract

Obeticholic acid (OCA) and elafibranor (ELA) are selective and potent agonists for the farnesoid X receptor (FXR) and dual peroxisome proliferator-activated receptor α/δ (PPAR-α/δ), respectively. Both agents have demonstrated clinical efficacy in nonalcoholic steatohepatitis (NASH). The present study used OCA and ELA to compare the effects of mono- and combination therapies on metabolic and histological endpoints in *Lep*^*ob/ob*^ mice with established diet-induced and biopsy-confirmed NASH (*ob/ob*-NASH). *ob/ob*-NASH mice were fed the AMLN diet high in trans-fat, fructose and cholesterol for 15 weeks, whereafter they received vehicle, OCA (30 mg/kg, PO, QD), ELA (3, 10 mg/kg, PO, QD), or combinations (OCA + ELA) for eight weeks. Within-subject comparisons were performed on histomorphometric changes, including fractional area of liver fat, galectin-3 and Col1a1. OCA and ELA monotherapies improved all quantitative histopathological parameters and OCA + ELA combinations exerted additive effects on metabolic and histological endpoints. In agreement with their different molecular mechanisms of action, OCA and ELA monotherapies elicited distinct hepatic gene expression profiles and their combination led to profound transcriptome changes associated with further improvements in lipid handling and insulin signaling, suppression of immune responses and reduced extracellular matrix formation. In conclusion, these findings provide preclinical proof-of-concept for combined FXR and PPAR-α/δ agonist-based therapies in NASH.

## Introduction

Non-alcoholic fatty liver disease (NAFLD) is a complex disease spectrum ranging from simple steatosis with a relatively benign course to its more aggressive manifestation, non-alcoholic steatohepatitis (NASH), defined by histopathological hallmarks following liver biopsy. NASH carries a poor prognosis and can progress to cirrhosis, hepatocellular cancer and end-stage liver disease^1,2^. NAFLD is closely associated with the metabolic syndrome and the globalization of NAFLD runs in close parallel to the epidemics of obesity and type 2 diabetes^3–5^. The NASH pathogenesis involves multiple drivers associated with aberrant fat and carbohydrate handling, including formation of fatty acid-derived lipotoxic species, accumulating mitochondrial oxidative stress, reduced insulin sensitivity and imbalanced immune responses. A gradual exhaustion of hepatocyte regenerative capacity may also lead to progressive hepatocellular injury, disrupted hepatic cytoarchitecture and excess extracellular matrix formation leading to cirrhosis and development of hepatocellular carcinoma^6–8^.

As the extent of liver fibrosis is the major driver of cardiovascular co-morbidity, malignancy and mortality in NASH^9–11^, there is a major emphasis on developing anti-fibrotic therapies for the disease. The current understanding of the molecular mechanisms triggering the onset and progression of NASH has led to leveraging multiple targets in various stages of clinical development. To date, clinical development programs have largely evaluated single drug treatments for NASH^12,13^. However, given the multifactorial molecular mechanisms that drive progression of the disease, future therapies are likely to involve drug combination regimens which incorporate complementary molecular modes of action to enhance NASH resolution and promote fibrosis regression. Accordingly, drug combinations have now advanced into clinical studies for NASH (ClinicalTrials.gov; NCT03449446, NCT03517540, NCT03776175).

Promising approaches currently in late stage clinical development include agonists specific for the farnesoid X receptor (FXR) and peroxisome proliferator-activated (PPAR) receptor subtypes. Obeticholic acid (OCA) is a first-in-class selective FXR agonist with approximately 100-fold greater potency than its natural bile acid homologue^14^, and is currently approved in combination with ursodeoxycholic acid (UDCA) in adults with an inadequate response to UDCA, or as monotherapy in adults unable to tolerate UDCA for the treatment of primary biliary cholangitis (PBC)^15,16^. OCA was initially demonstrated to increase insulin sensitivity and improve liver functional markers in patients with type 2 diabetes and NAFLD^17^. These findings have been extended in the FLINT study, a larger phase 2 trial in NASH patients, reporting significant improvements in liver histopathology, including a decrease in liver fibrosis^18^. The selective dual PPAR-α/δ agonist, elafibranor (ELA, also known as GFT-505), has shown similar beneficial effects on liver function tests and histopathology in NASH patients^19^. These findings have been corroborated in various diet-induced obese mouse models of NASH^20–25^.

FXR serves as primary sensor for regulating enterohepatic bile acid flow and metabolism. FXR is highly expressed in the liver, intestine and adipose tissue^26^, and controls several gene expression programs associated with cholesterol metabolism, lipogenesis, gluconeogenesis and glycogenolysis, promoting increased fatty acid oxidation and triglyceride clearance as well as reduced glucose production^27,28^. In addition, FXR-induced anti-inflammatory effects have been linked to direct inhibition of inflammatory signaling cascades in hepatocytes and monocytes as well as to improved integrity of the intestinal barrier^29–31^. PPARs are prominent fatty acid sensors controlling lipid and carbohydrate metabolism in the liver (PPAR-α, PPAR-β/δ), muscle (PPAR-β/δ) and adipose tissues (PPAR-δ/γ), being master transcriptional regulators of hepatic fatty acid utilization, adipogenesis and peripheral insulin sensitivity^32,33^.

The complementary and overlapping transcriptional pathways controlled by FXR and PPARs prompted us to investigate whether combined FXR and PPAR agonism could promote additive benefits on metabolic and histological outcomes in NASH. The present study therefore aimed to assess the therapeutic efficacy of OCA and ELA monotherapies in comparison to OCA and ELA co-treatment in an *ob/ob* mouse model of high-fat/carbohydrate diet-induced and biopsy-confirmed NASH.

## Materials and Methods

### Animals

The Danish Animal Experiments Inspectorate approved all experiments which were conducted using internationally accepted principles for the use of laboratory animals under the personal license #2013-15-2934-00784. B6.V-Lep^ob^/JRj (*ob/ob*) mice (6 weeks old) were from Janvier Labs (Le Genest Saint Isle, France) and housed in a controlled environment (12 h light/dark cycle, lights on at 3 AM, 21 ± 2 °C, humidity 50 ± 10%). Each animal was identified by an implantable subcutaneous microchip (PetID Microchip, E-vet, Haderslev, Denmark). Mice had *ad libitum* access to tap water and a diet high in fat (40%, containing 18% trans-fat; 40% carbohydrates, 20% fructose) and 2% cholesterol (AMLN diet; D09100301, Research Diets, New Brunswick, NJ)^34,35^ for 15 weeks prior to treatment start and during drug treatment. All *ob/ob* animals underwent liver biopsy prior to treatment (see below), whereupon they were single-housed throughout the remainder of the study. An outline of the study design is shown in Supplementary Figure S1.

### Baseline liver biopsy

The biopsy procedure was applied to all mice approximately three weeks before completion of the dieting period, as detailed previously^34^. In brief, mice were pretreated with enrofloxazin (Baytril®, 5 mg/mL, 1 mL/kg; Bayer, Leverkusen, Germany) one day prior to biopsy. On the surgery day, mice were anesthetized with isoflurane (2–3%, in 100% oxygen), a small abdominal incision in the midline was made, and the left lateral lobe of the liver was exposed. A cone-shaped wedge of liver tissue (50–100 mg) was excised from the distal part of the lobe. The cut surface of the liver was closed by electrosurgical bipolar coagulation using an electrosurgical unit (ERBE VIO 100 C, ERBE, Marietta, GA). The liver was returned to the abdominal cavity, the abdominal wall was sutured and skin stapled. Carprofen (Rimadyl®, 5 mg/mL, 0.01 mL/10 g; Pfizer, NY) and enrofloxazin (5 mg/mL, 1 mL/kg, i.p.) were administered at the time of surgery and at post-operative day one and two. Animals were single-housed after the procedure and recovered for three weeks prior to drug treatment.

### Drug treatment

OCA (Intercept Pharmaceuticals, New York, NY) and ELA (Genfit, Cambridge, MA) were freshly dissolved in 0.5% carboxymethyl cellulose and orally administered in a dosing volume of 2.5 ml/kg. Animals were stratified (n = 9–12 per group) based on mean fibrosis as assessed by collagen 1a1 (Col1a1) immunostaining (primary) and body weight (secondary). After 15 weeks on AMLN diet, mice were treated once daily for 8 weeks with vehicle, OCA (30 mg/kg), ELA (3 or 10 mg/kg), OCA (30 mg/kg) + ELA (3 mg/kg), OCA (30 mg/kg) + ELA (10 mg/kg). To control for the two simultaneous drug injections, a vehicle injection was administered after monotherapy. Food intake (24 h) was measured once weekly. A terminal blood sample was collected from the tail vein in non-fasted mice and used for plasma biochemistry. Animals were sacrificed by cardiac puncture under isoflurane anesthesia. Liver samples were processed as described below.

### Body weight and body composition analysis

Body weight was monitored daily during the intervention period. Whole-body fat and lean mass was analyzed in week 7 of the treatment period by non-invasive EchoMRI scanning using EchoMRI-900 (EchoMRI, Houston, TX). During the scanning procedure, mice were placed in a restrainer for 90–120 s.

### Plasma biochemistry

Terminal plasma concentrations of total triglycerides (TG), total cholesterol (TC), alanine aminotransferase (ALT) and aspartate aminotransferase (AST) were determined as described previously^34^.

### Liver histology and digital image analysis

Baseline liver biopsy and terminal samples (both from the left lateral lobe) were fixed overnight in 4% paraformaldehyde. Liver tissue was paraffin-embedded and sectioned (3 µm thickness). Sections were stained with hematoxylin-eosin (HE), anti-galectin-3 (cat. 125402, Biolegend, San Diego, CA), or anti-type I collagen (Col1a1, cat. 1310-01, Southern Biotech, Birmingham, AL) using standard procedures^34^, and quantitative histomorphometry was applied using a digital imaging software (Visiomorph®, Visiopharm, Hørsholm, Denmark). The fractional area of liver fat (macrosteatosis) was determined on HE-stained sections and expressed relative to total sectional area. The fractional area of galectin-3 and Col1a1 immunostaining was expressed relative to total parenchymal area by subtracting corresponding fat area determined on adjacent HE-stained sections. All histological assessments were performed by histologists blinded to the experimental groups.

### RNA sequencing

Liver transcriptome analysis was performed by RNA sequencing on RNA extracts from terminal liver samples (15 mg fresh tissue), as described in detail elsewhere^34^. The RNA quantity was measured using Qubit® (Thermo Scientific, Eugene, OR). The RNA quality was determined using a bioanalyzer with RNA 6000 Nano kit (Agilent, Waldbronn, Germany). RNA sequence libraries were prepared with NeoPrep (Illumina, San Diego, CA) using Illumina TruSeq stranded mRNA Library kit for NeoPrep (Illumina, San Diego, CA) and sequenced on the NextSeq. 500 (Illumina, San Diego, CA) with NSQ 500 hi-Output KT v2 (75 CYS, Illumina, San Diego, CA). Reads were aligned to the GRCm38 v84 Ensembl Mus musculus genome using STAR v.2.5.2a with default parameters^36^. Differential gene expression analysis was performed with DEseq2^37^. Genes with a Benjamini and Hochberg adjusted P ≤ 0.05 (5% False Discovery Rate, FDR) were regarded as statistically significantly regulated.

### Functional annotation of differentially expressed genes

Candidate NAFLD- and fibrosis-associated pathways were used to annotate genes involved in disease progression (Supplementary Table S1). A gene set analysis was conducted with the R package PIANO version 1.18.1 using the Stouffer method, and p-values were corrected for multiple testing using the Benjamini-Hochberg method (FDR < 0.05). The Reactome pathway database was retrieved and used for gene annotation enrichment analysis.

### Statistical analyses

Except from RNA sequencing, data were analyzed using GraphPad Prism v7.03 software (GraphPad, La Jolla, CA). All results are shown as mean ± standard error of mean (S.E.M.). A two-way ANOVA with Tukey’s multiple comparisons test was performed for body weight and quantitative histological analyses. A one-way ANOVA with Dunnett’s post-hoc test was used for all other parameters. A p-value < 0.05 was considered statistically significant.

## Results

### Effects of OCA and ELA treatment on body weight and whole-body fat mass

**Figure 1:**
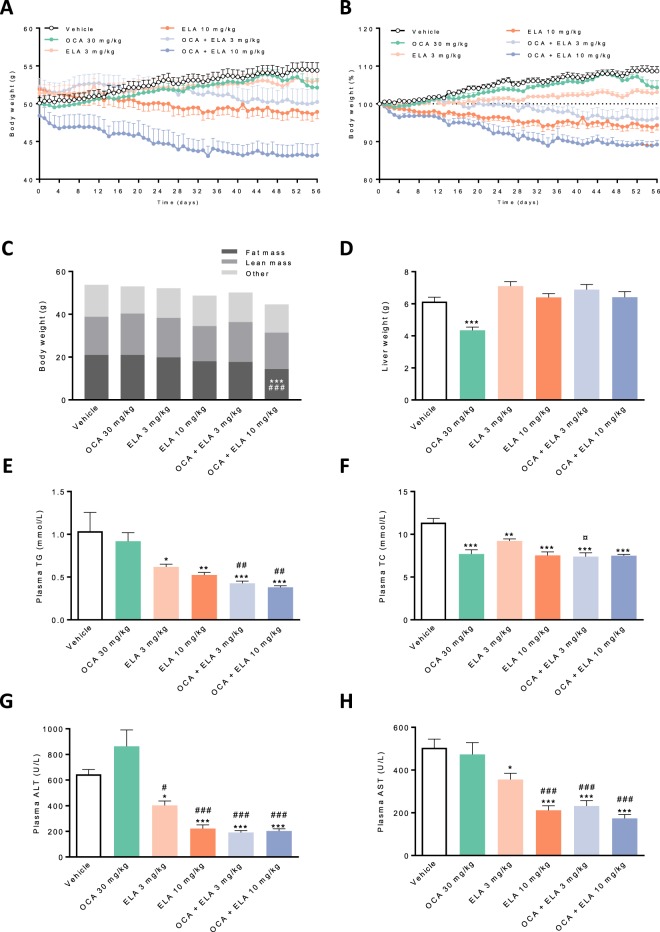
Combined OCA and ELA treatment improves metabolic markers in AMLN *ob/ob*-NASH mice with biopsy-confirmed liver pathology. (**A**) Body weight (g), (**B**) Body weight gain relative to treatment start (day 0), (**C**) Whole-body composition, (**D**) Terminal liver weight; (**E**) Plasma total triglycerides (TG); (**F**) Plasma total cholesterol (TC); (**G**) Plasma alanine aminotransferase (ALT); (**H**) Plasma aspartate aminotransferase (AST).

Body weight in vehicle-dosed AMLN *ob/ob*-NASH mice gradually increased over the 8-week dosing period, resulting in an endpoint body weight of 54.5 ± 0.9 g, equivalent to a weight gain of 8.7 ± 1.2% relative to dosing start (Fig. 1A,B). When administered alone, OCA 30 mg/kg did not influence weight gain in AMLN *ob/ob*-NASH mice (52.1 ± 1.2 g, body weight gain 4.3 ± 1.8%). In contrast, ELA 10 mg/kg (48.9 ± 0.9 g, −5.7 ± 1.3%, p < 0.05 vs. vehicle), but not ELA 3 mg/kg (53.1 ± 1.4 g, 3.1 ± 0.9%), significantly reduced endpoint body weight compared to vehicle controls. Co-administration of OCA + ELA 3–10 mg/kg promoted further weight loss compared to both vehicle control and monotherapy (OCA + ELA 3 mg/kg: 50.1 ± 1.9 g, −3.8 ± 2.8%, p < 0.001 vs. vehicle; OCA + ELA 10 mg/kg: 43.4 ± 1.6 g, −10.8 ± 1.1%, p < 0.001 vs. vehicle). Treatments did not affect food intake (Supplementary Figure S2). OCA + ELA 10 mg/kg, but not individual monotherapies, also significantly reduced whole-body fat mass in AMLN *ob/ob*-NASH mice (p < 0.001 vs. vehicle). Treatments had no effect on lean tissue mass (Fig. 1C). Only OCA significantly reduced hepatomegaly in AMLN *ob/ob*-NASH mice (p < 0.001, Fig. 1D).

### OCA and ELA treatment improve plasma biochemical markers

ELA and OCA + ELA, but not OCA alone, resulted in significantly reduced TG levels (Fig. 1E). Combination treatment did not lead to further reductions in TC levels compared to OCA and ELA treatment alone (Fig. 1F). OCA did not affect ALT and AST levels, whereas ELA 10 mg/kg, OCA + ELA 3 mg/kg, and OCA + ELA 10 mg/kg resulted in similar significant reductions in ALT and AST concentrations (Fig. 1I,H).

### OCA and ELA exert additive effects to reduce steatosis and inflammation

**Figure 2:**
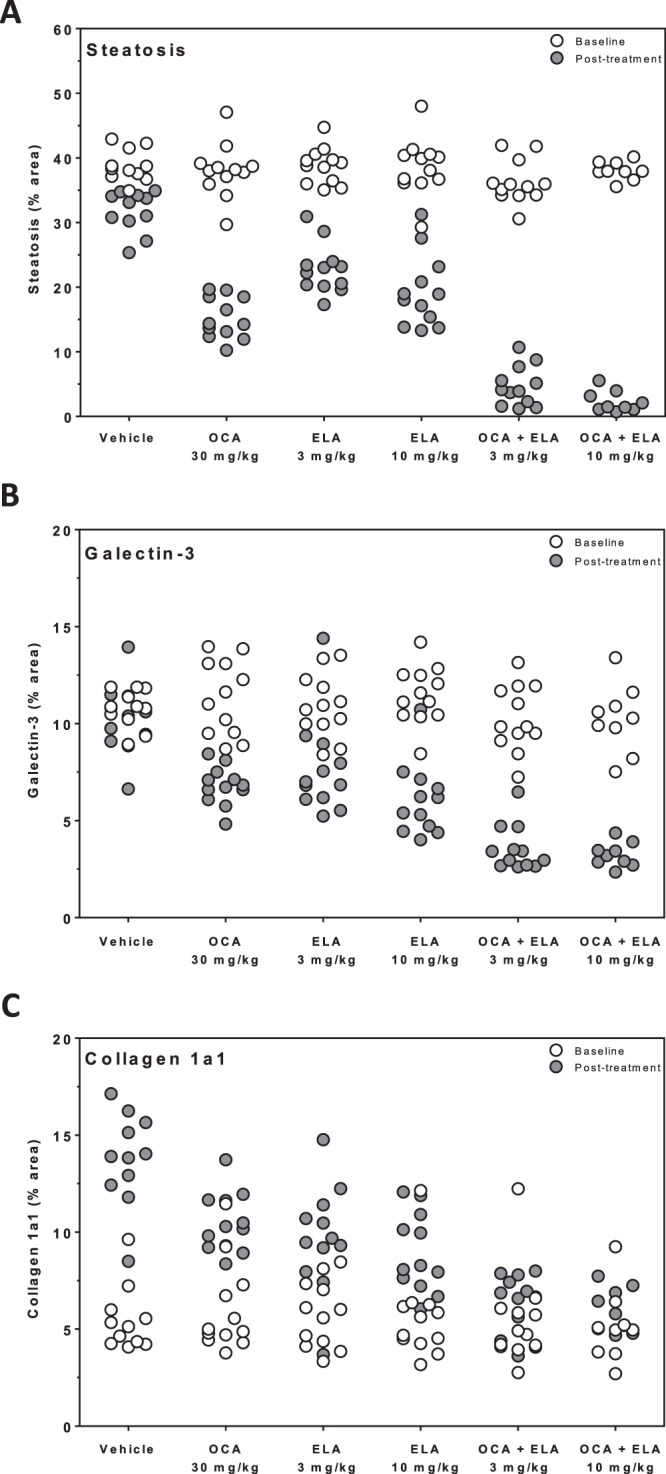
Combined OCA and ELA treatment promotes additive effects on quantitative liver histopathology in AMLN *ob/ob*-NASH mice. Liver histomorphometric data are indicated before (baseline) and after treatment. (**A**) Fractional area (%) of fat; (**B**) Galectin-3; (**C**) Collagen 1a1. Data are expressed as % of total parenchymal area (subtraction of fat area). See Table 1 for corresponding numerical data and statistical evaluation.

Average baseline (prebiopsy) fractional (%) area of liver fat (36.3–38.8%, p = 0.435, Fig. 2A) and galectin-3 staining (10.3–11.5%, p = 0.366, Fig. 2B) were similar across all experimental groups (Table 1). Mean %-area of fat was significantly reduced after treatment with OCA 30 mg/kg (15.2 ± 0.9%, p < 0.001), amounting to a 60% reduction compared to baseline (Table 1). ELA treatment promoted almost similar reductions in the proportionate area of fat (3 mg/kg, 22.8 ± 1.1%; 10 mg/kg, 19.3 ± 1.6%, p < 0.001). Further improvements in steatosis were achieved with co-administration of OCA + ELA, with both doses of ELA showing equal efficacy (3 mg/kg, 4.7 ± 0.9%; 10 mg/kg, 2.3 ± 0.5%, p < 0.001 vs. vehicle), resulting in >90% reduction in fat area-% compared to baseline (Table 1). *ob/ob*-NASH control mice did not show further progression in %-area of galectin-3 immunostaining during the vehicle dosing period. Compared to vehicle controls, %-area of galectin-3 was equally reduced following treatment with OCA 30 mg/kg (6.8 ± 0.3%, p < 0.01) and ELA (3 mg/kg, 7.7 ± 0.7%, p < 0.01; 10 mg/kg, 6.1 ± 0.5%, p < 0.001), see Fig. 2A and Table 1. Further lowering of galectin-3%-area was observed after combined treatment with OCA + ELA 3 mg/kg (3.6 ± 0.3%, p < 0.001) and OCA + ELA 10 mg/kg (3.2 ± 0.2%, p < 0.001), see Fig. 2B and Table 1. Representative photomicrographs on terminal liver fat and galectin-3 stainings are shown in Fig. 4.

**Figure 3:**
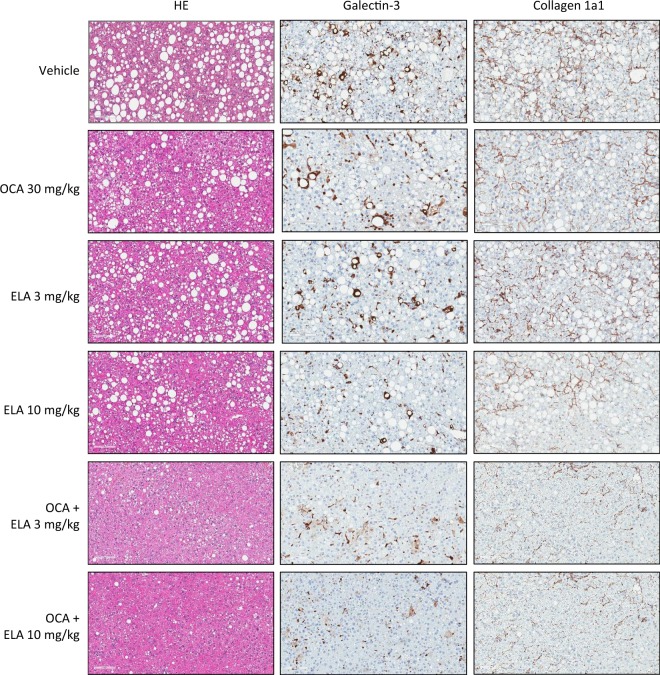
Multivariate visualization of the anti-steatotic, anti-inflammatory, and anti-fibrotic effects of combined OCA and ELA treatment in AMLN *ob/ob*-NASH mice. Terminal liver histomorphometric data of fractional area (%) of collagen 1a1 plotted against corresponding fractional area (%) of steatosis (panel A, r^2^ = 0.54) and galectin-3 (panel B, r^2^ = 0.60), respectively. Linear regression lines are indicated.

**Table 1:** Combined OCA and ELA administration promotes additive treatment effects to improve quantitative liver histopathology in AMLN *ob/ob*-NASH mice. Fractional area (% ± S.E.M.) at the end of study; ^###^p < 0.001 vs. before treatment (paired t-test); *p < 0.05 vs. vehicle, ^O^p < 0.05 vs. OCA alone, ^E^p<0.05 vs. corresponding ELA dose alone (two-way ANOVA, Bonferroni’s post-hoc test).

Treatment	Lipid (steatosis)		Galectin-3 (inflammation)		Collagen 1a1 (fibrosis)	
	% Fractional area					
	Before treatment	After treatment	Before treatment	After treatment	Before treatment	After treatment
Vehicle (n = 11)	38.8 ± 0.7	31.8 ± 1.0	10.8 ± 0.3	10.2 ± 0.6	5.5 ± 0.5	13.8 ± 0.7^###^
OCA 30 mg/kg (n = 12)	38.0 ± 1.2	15.2 ± 0.9*	11.3 ± 0.6	6.8 ± 0.3*	6.0 ± 0.7	10.5 ± 0.4*
ELA 3 mg/kg (n = 11)	38.8 ± 0.8	22.8 ± 1.1*	10.9 ± 0.5	7.7 ± 0.7*	5.8 ± 0.5	9.7 ± 0.8*
ELA 10 mg/kg (n = 12)	38.6 ± 1.3	19.3 ± 1.6*	11.5 ± 0.4	6.1 ± 0.5*	5.6 ± 0.7	8.9 ± 0.6*
OCA + ELA 3 mg/kg (n = 12)	36.3 ± 1.0	4.7 ± 0.9*^O^^E^	10.3 ± 0.5	3.6 ± 0.3*^OE^	5.4 ± 0.7	6.3 ± 0.4*^OE^
OCA + ELA 10 mg/kg (n = 9)	38.1 ± 0.5	2.3 ± 0.5*^OE^	10.3 ± 0.6	3.2 ± 0.2*^OE^	5.1 ± 0.6	6.0 ± 0.4*^OE^

**Figure 4:**
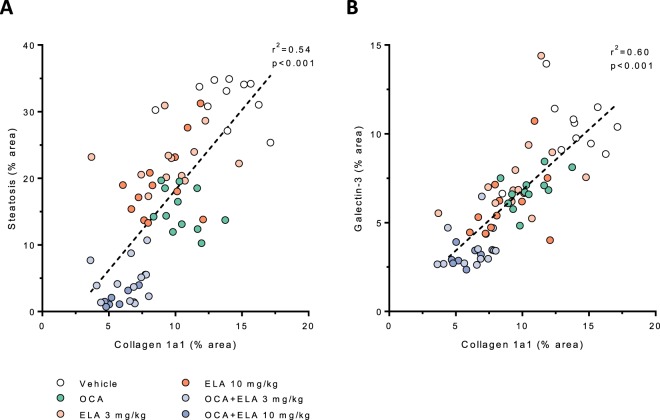
Representative histological effects of OCA and ELA monotherapy vs. combined treatments in AMLN *ob/ob*-NASH mice. Representative photomicrographs depicting the anti-steatotic (HE staining), anti-inflammatory (Galectin-3 immunostaining), and anti-fibrotic (Collagen 1a1 immunostaining) effects of OCA and ELA monotherapy vs. combined treatment in AMLN *ob/ob*-NASH mice.

### OCA and ELA exert additive effects to slow fibrosis progression

Average baseline proportionate area of liver Col1a1 immunostaining was similar (5.1–6.0%, p = 0.953) in all experimental groups (Fig. 2C, Table 1). In vehicle controls, Col1a1%-area increased from 5.5 ± 0.5% to 13.8 ± 0.7% (p < 0.001, paired t-test) over the 8-week dosing period (Fig. 2C, Table 1). Compared to vehicle controls, increases in Col1a1 area were less pronounced in AMLN *ob/ob*-NASH mice receiving OCA 30 mg/kg (10.5 ± 0.4%, p < 0.01), ELA 3 mg/kg (9.7 ± 0.8%, p < 0.001), or ELA 10 mg/kg (8.9 ± 0.6%, p < 0.001) treatment. Both OCA + ELA 3 mg/kg (6.3 ± 0.4, p < 0.001 vs. vehicle) and OCA + ELA 10 mg/kg (6.0 ± 0.4, p < 0.001 vs. vehicle) effectively prevented further increases in Col1a1%-area in AMLN *ob/ob*-NASH mice, see Fig. 2C and Table 1. Individual reductions in Col1a1 fractional area closely correlated to corresponding improvements in steatosis and galectin-3%-area (Fig. 3). Representative photomicrographs on post-biopsy Col1a1 immunostaining are shown in Fig. 4. In addition to quantitative histology, liver histomorphology was evaluated in pre- vs. post-treatment liver biopsies, as outlined by Kleiner *et al*.^38^. Individual histopathology scores are indicated in Supplementary Figs S3 and S4. At baseline, AMLN *ob/ob*-NASH mice showed severe steatosis (score 3), mild-to-moderate lobular inflammation (score 1–2) and a relatively low rate of hepatocyte ballooning. Baseline fibrosis stage was mild to moderate (F1-F2) in all treatment groups. Whereas NAFLD activity scores (NAS) were unchanged or slightly increased over the 8-week dosing period in AMLN *ob/ob*-NASH vehicle control mice, treatment with OCA + ELA 3 mg/kg and OCA + ELA 10 mg/kg resulted in significant reductions in NAS, mainly due to reduced steatosis and inflammation scores. Drug treatments did not significantly change fibrosis scores compared to vehicle controls.

### OCA and ELA exert additive effects on disease-associated molecular pathways

**Figure 5:**
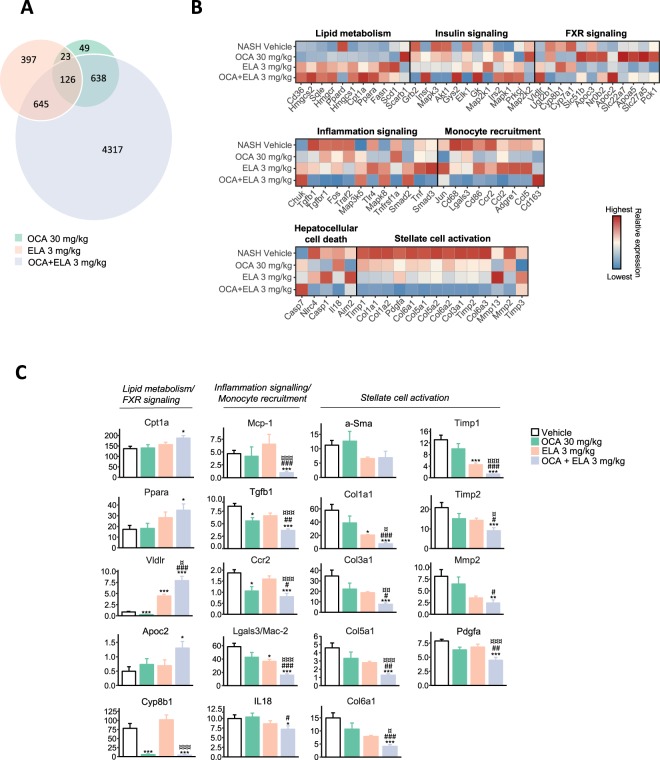
OCA and ELA exert additive effects on hepatic global gene expression and NAFLD/fibrosis-associated candidate genes in AMLN *ob/ob*-NASH mice. (**A**) Venn diagram depicting the total number of shared and separate differentially expressed genes (DEGs) in AMLN *ob/ob*-NASH mice receiving OCA (30 mg/kg), ELA (3 mg/kg), or combined OCA (30 mg/kg) + ELA (3 mg/kg) treatment as compared to vehicle controls; (**B**) Relative gene expression levels (z-scores) of differentially expressed candidate genes associated with NASH and fibrosis (see Supplementary Table S1 for complete list of candidate genes); (**C**) Expression levels (RPKM values) of genes represented in most significantly regulated pathways associated with lipid metabolism/FXR signaling, monocyte recruitment/inflammation signaling, and stellate cell activation/extracellular matrix (ECM) organization. *p < 0.05, **p < 0.01, ***p < 0.001 (vs. vehicle); ^#^p < 0.05, ^##^p < 0.01, ^###^p < 0.01 (vs. OCA alone); ^¤^p < 0.01, ^¤¤^p < 0.01, ^¤¤¤^p < 0.001 (vs. Elafibranor alone).

**Figure 6:**
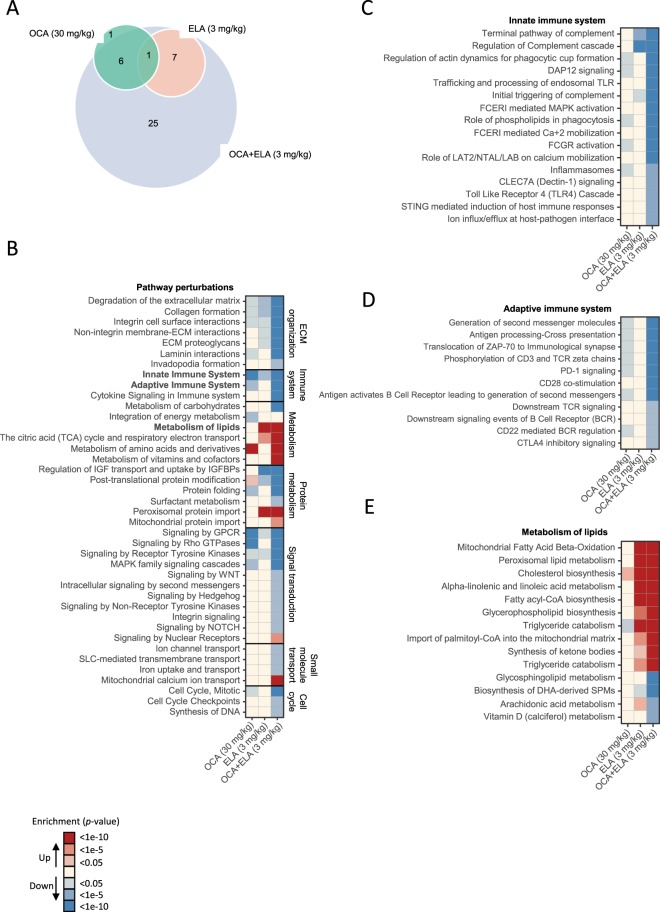
Global analysis of pathway perturbations following combined OCA and ELA treatment in AMLN *ob/ob*-NASH mice. (**A**) Venn diagram depicting the total number of shared and separate Reactome pathway perturbations in *ob/ob*-NASH mice receiving OCA (30 mg/kg), ELA (3 mg/kg), or combined OCA (30 mg/kg) + ELA (3 mg/kg) treatment as compared to vehicle controls; (**B**) Significantly regulated Reactome pathways grouped according to biological pathway. Further resolution of selected Reactome pathways, including (**C**) Innate immune system, (**D**) Adaptive immune system, and (**C**) Metabolism of lipids. Upregulated (red color gradient) and downregulated (blue color gradient) gene expression in individual Reactome pathways are ranked according to statistical significance level.

The effect on hepatic global gene expression was characterized in mice receiving OCA 30 mg/kg, ELA 3 mg/kg, and OCA + ELA 3 mg/kg, respectively. Differentially expressed genes (DEG) were determined with reference to vehicle controls. OCA and ELA monotherapies promoted global gene expression signatures with a limited overlap in differentially expressed genes (DEGs). OCA + ELA treatment resulted in a markedly higher number (n = 5,726) of DEGs compared to OCA (n = 836) and ELA (n = 1,191) alone (Fig. 5A). For initial delineation of DEGs identified, we probed for NAFLD and fibrosis-associated candidate genes (Supplementary Table S1). OCA and ELA monotreatment resulted in transcriptional regulations across all pre-defined candidate gene sets. Notably, combined OCA + ELA treatment resulted in extensive perturbations compared to individual mono-treatments (Fig. 5B), and showed significant additive effects on the expression of several transcriptional markers associated with increased lipid and cholesterol metabolism (*Cpt1a, Ppara, Vldlr, Apoc2*), decreased immune cell function (*Mcp-1, Ccr2, Lgals3, IL18*), as well as reduced collagen formation (*Col1a1, Col3a1, Col5a1, Col6a1*) and extracellular matrix (ECM) remodeling (*Timp1, Timp2, Mmp2, Pdgfa, Tgfb*), see Fig. 5C. To improve data resolution, gene annotation maps were generated using the Reactome pathway database. The unsupervised gene set enrichment analysis revealed that combined OCA and ELA treatment promoted additive modulatory effects on multiple metabolic and immune signaling pathways (Fig. 6A) concurrent with suppression of a considerable number of additional genes involved in ECM remodeling (*Adam8, CapnsS1, Cd44, Col4a1/2, Col5a2, Col6a2/3, Col8a1, Ctsb, Ctss, Fblim1, Fbn2, Icam1, Itgb2, Loxl2/3, Ltbp2, Mfap2, Mmp12/14, Nid1, Pdfgb, Prkca, Rsu1, Serpinh1, Sparc, Tgfb1/2, Thbs1*), see Fig. 6B. A further analysis of selected Reactome pathways indicated significant inhibitory effects of OCA and ELA co-administration on both the innate (including *C6, C9, C1s1, C3ar1, Cd68, Rap2b, Tlr1, Tlr4)* and adaptive (including *Actr1b, Cd4, Cd74, Cd81, Cd86, Hcst, Mrc2, Ncf2, Trem2*) immune system (Fig. 6C,D). Stimulated hepatic fatty acid transport and utilization following ELA and OCA + ELA treatment was suggested by upregulation of gene transcriptional programs linked to *e.g*. fatty acid β-oxidation/peroxisomal lipid metabolism/triglyceride catabolism (including *Acaa1a, Acot4, Acox1, Amacr, Cidea, Cpt1a, Crat, Ehhadh, Ppard, Pnpla2*), see Fig. 6E. Genes encoding enzymes associated with oxidative stress (including *Alox, Cox, Cyp2, Mpo, Nox*) and antioxidant function (including *Cat, Gsr, Gst, Prdx, Sod*) were not affected by treatments.

## Discussion

The present study characterized the combined effect of eight weeks of OCA and ELA treatment in an AMLN diet-induced obese *ob/ob* mouse model of biopsy-confirmed NASH. Monotherapy with OCA or ELA improved all liver quantitative histopathological parameters and drug treatment combinations exerted significantly greater effects than either monotherapy on both liver histopathology and associated liver transcriptome changes.

As in NASH patients, mouse models of high-fat/carbohydrate diet-induced NASH show heterogeneous baseline disease state severity and rates of progression^12,20,22,34,39^. To account for the inherent variability in histopathological parameters, AMLN *ob/ob-*NASH mice were stratified and randomized to treatment based on biopsy-confirmed liver histopathology which also enabled assessment of within-subject treatment responses at study termination. Consistent with previous findings in the model, quantitative histomorphometry on liver pre-biopsies indicated marked liver fat accumulation, significant inflammation and collagen deposition in AMLN *ob/ob*-NASH mice^34,35,39,40^. Compared to baseline, *ob/ob*-NASH control mice showed significantly increased terminal fractional area of Col1a1, being in agreement with a recent report using this model^21^. Similar to previous studies in AMLN *ob/ob*-NASH mice^20,21^, galectin-3 immunohistochemistry was applied for quantitative assessment of hepatic inflammation. Galectin-3 (also known as Mac-2) is expressed in various immunocompetent and inflammatory cells, in particular activated macrophages, but also in eosinophils, mast cells and activated lymphocyte profiles^41,42^. In addition to a prominent role in inflammation, galectin-3 secretion from immune cells stimulates proliferation and activation of myofibroblasts, including hepatic stellate cells (HSCs)^42,43^. It should be noted that activated HSCs also express galectin-3, as demonstrated in cultured primary HSCs^44–47^ as well as in chemotoxin- and surgically-induced rodent models of severe fibrotic liver injury^45,46^. Although it is not resolved if activated HCSs could potentially contribute to galectin-3 expression in NASH models with less advanced inflammation and fibrosis, liver samples from AMLN *ob/ob*-NASH mice show highly different distribution of α-SMA and galectin-3 immunostaining (data not shown), which argues for HSC-derived galectin-3 expression being neglectable in this model.

OCA and ELA were probed for individual and combined drug effects on quantitative liver histopathology, plasma biochemistry, body weight and body composition in AMLN *ob/ob*-NASH mice. Individual drug doses were selected based on previous studies characterizing effects of OCA and ELA monotherapy in AMLN *ob/ob*-NASH mice^20,21,40^. OCA treatment resulted in significant quantitative reductions in liver fat, inflammation and collagen deposition. The marked improvement in steatosis was reflected by reduced hepatomegaly in OCA-treated mice. OCA was weight neutral and did not influence body weight and whole-body fat mass in AMLN *ob/ob*-NASH mice, indicating that OCA-induced improvements in liver histopathology were not dependent on changes in adiposity. These findings corroborate recently reported histological and metabolic effects of OCA treatment in AMLN *ob/ob*-NASH mice^20,21,40^ as well as in a comparable AMLN diet-induced obese (DIO) NASH model in C57BL/6 J mice (AMLN DIO-NASH)^20,23^. Also, our data are in line with previous data on metabolic, anti-inflammatory and anti-fibrotic effects of FXR agonist treatment, including OCA, in other rodent models of obesity with features of simple steatosis/low-grade inflammation^22,48^ and NASH^49^, as well as in nutrient-deficient dietary^50–52^ and surgery-based models of NASH^51^. It should be noted that others have reported concomitant reductions in body weight and white adipose tissue mass following administration of OCA in high-caloric diet-fed mice with simple steatosis^25^, suggesting model phenotype-specific effects of OCA on adipose tissue metabolism. Several genes involved in triglyceride and cholesterol metabolism are major transcriptional targets of FXRs and act in concert to regulate hepatocyte lipid clearance^53,54^, and suppression of carbohydrate-responsive gene expression and improved hepatic insulin resistance may contribute to FXR-mediated lowering of hepatic lipogenesis^55^. FXRs activation also exerts immunosuppressive actions in various immune cell populations, including monocytes and macrophages^56–59^, and inhibited pro-fibrotic activity of hepatic stellate cells^14^.

Our data also confirm and extend upon findings of ELA-induced reductions in body weight, liver histomorphometry and plasma biochemical markers in AMLN *ob/ob*-NASH mice^20^. Interestingly, ELA treatment reduced weight gain without influencing hepatomegaly in AMLN *ob/ob*-NASH mice. Unlike humans, stimulated PPAR-α and PPAR-δ function has been associated with weight loss and appetite suppression in diet-induced obese mice^60,61^. In addition, PPAR-α agonist-mediated hepatocyte peroxisome proliferation can lead to rodent-specific increases in liver mass^62,63^, which is therefore likely to prevent ELA from improving hepatomegaly in rodent models of NASH^20,25^. Compared to OCA, the effect of ELA treatment has been less characterized in animal models of NASH. Previous reports have demonstrated that ELA ameliorates steatosis, inflammation, and fibrosis in AMLN DIO-NASH mice^20^, improves liver metabolic parameters and reverses development of fibrosis in nutrient-deficient and hepatotoxin-induced models of NASH^25^. The significant reduction of steatosis in ELA-treated AMLN *ob/ob*-NASH mice is consistent with the diverse actions of PPAR-α on lipid metabolic signaling pathways. The major function of PPAR-α is to facilitate hepatic fatty acid utilization by transcriptional upregulation of rate-limiting peroxisomal and mitochondrial enzymes controlling fatty acid transport and β-oxidation^64^, mitochondrial ketogenesis^65^ as well as lipolysis^66^. Also, selective PPAR-α agonists stimulate hepatic clearance of triglyceride-rich lipoproteins^67^. Because rodent liver PPAR-α expression is mainly confined to hepatocytes^68,69^, the anti-inflammatory and anti-fibrotic effects of PPAR-α agonists are considered subsequent to direct actions on liver parenchymal cells preventing the release of lipotoxic and pro-inflammatory mediators^70^. Interestingly, the beneficial effects of ELA on steatohepatitis are preserved in high-fat diet-fed double transgenic hApoE2/PPAR-α knock-out mice^25^, indirectly supporting the concept that PPAR-δ (also termed PPAR-β/δ) contributes to the anti-NASH effects of ELA. PPAR-δ agonist-mediated actions on lipid metabolism is reportedly dependent on co-operative PPAR-α activity^71^, and may indirectly reflect suppression of hepatic gluconeogenesis and hepatic glucose output^72,73^. Whereas PPAR-δ regulates Kupffer cell polarization towards an M2 anti-inflammatory phenotype^74^, functional implications of PPAR-δ expression during hepatic stellate cell activation remain to be clarified^75^.

We have previously reported that AMLN *ob/ob*-NASH and DIO-NASH mice display hypercholesterolemia, but not hypertriglyceridemia, concurrent with marked hepatic accumulation of triglycerides and cholesterol^20,34,39,76,77^. This is also a characteristic of other cholesterol-enriched Western diet-based mouse models of NASH^76,77^, and it is speculated that increased dietary cholesterol intake may impair hepatocyte triglyceride secretion by modifying cholesterol ester and lipoprotein synthesis^77,78^. As OCA and ELA mono-treatment, as well as combinations, reduced plasma total triglyceride and cholesterol levels while also upregulating hepatic genes linked to cholesterol biosynthesis, this further points to complex regulation of cholesterol metabolism in AMLN *ob/ob*-NASH mice. The lack of hypertriglyceridemia in AMLN *ob/ob*-NASH mice contrasts the dyslipidemia profile in NAFLD/NASH patients which is characterized by elevated triglyceride and low-density lipoprotein cholesterol levels as well as decreased high-density lipoprotein cholesterol concentrations^79,80^. Although we did not specifically determine the plasma lipoprotein cholesterol profile in the present study, it should be emphasized that DIO mice (and rats) are generally resistant to develop human-like atherogenic dyslipidemia^81,82^, which should be taken into account when interpreting changes in plasma lipid profiles in these models, including AMLN *ob/ob*-NASH mice.

Notably, OCA and ELA combinations exerted additive therapeutic effects on liver histology in AMLN *ob/ob*-NASH mice. Compared to baseline, co-administration of OCA and ELA led to marked improvements in the proportionate area of liver fat and galectin-3. The histomorphometric analyses indicated that OCA markedly enhanced the anti-fibrotic efficacy of both doses of ELA. Accordingly, combined treatment with OCA and ELA resulted in almost complete prevention of progressive hepatic Col1a1 deposition. The individual reduction in the proportionate area of Col1a1 was closely correlated with improvements in steatosis and inflammation, illustrating a highly consistent within-subject effect on all three histological endpoints. Also, AMLN *ob/ob*-NASH mice receiving both OCA and ELA treatment attained further weight loss as compared to that achieved by ELA alone. Whether the magnitude of weight loss contributed to promote further benefits on liver histopathology must await further studies using additional control conditions, *e.g*. weight-matched/calorie-restricted animals.

As discussed above, the enhanced liver histological outcome of combined OCA and ELA treatment is consistent with both receptor families being master transcriptional regulators of a broad group of metabolic enzymes and signaling molecules. Accordingly, our full-scale mapping of hepatic gene expression indicated that OCA and ELA monotherapies elicited distinct hepatic expression signatures in *ob/ob*-NASH mice and their combination led to profound changes in the liver transcriptome. The extent of transcriptional changes argues for widespread alterations in hepatic molecular signaling conferred by recruitment of complementary FXR and PPAR-α/δ associated mechanisms of action. Importantly, the liver transcriptome signature in *ob/ob*-NASH mice receiving combined OCA and ELA treatment supports the histological outcomes by indices of improved lipid handling and insulin signaling with concurrent attenuation of both immune and pro-fibrotic gene expression patterns. Interestingly, OCA and ELA co-administration resulted in a marked potentiation of their individual suppressive effects on a variety of pathways regulating the activity of the innate and adaptive immune system, suggesting that regression of steatohepatitis played an integral role in preventing further collagen deposition in AMLN *ob/ob*-NASH mice.

Histopathology scores partially correlated to the quantitative histological data, however, confirmed significantly reduced steatosis and inflammation in AMLN *ob/ob*-NASH mice receiving OCA monotherapy and the further improvements in these parameters following combined OCA and ELA treatment. The disparity likely reflects that histopathological disease scoring is based on semiquantitative morphological criteria and imaging-based quantitative histomorphometry may therefore better capture individual differences in treatment responses. The lack of effect of OCA on fibrosis scores is consistent with an 8-week dosing study in AMLN *ob/ob*-NASH mice^20^. Because 16 weeks of treatment with OCA has recently been reported effective in reducing both Col1a1 deposition and fibrosis scores in AMLN *ob/ob*-NASH mice^21^, this suggests that extended treatment periods may be required to observe parallel reductions in both parameters. Also, compared to the relative low doses of ELA (3–10 mg/kg/day) employed in the present study, a higher dose (30 mg/kg/day) has been reported to improve fibrosis scores in AMLN *ob/ob*-NASH mice^20^.

In conclusion, monotherapy with OCA and ELA improved histomorphometric indices of steatosis, inflammation and fibrosis in an *ob/ob* mouse model of biopsy-confirmed NASH. When administered in combination, OCA and ELA promoted additive metabolic and histological effects. Liver transcriptome changes indicated that OCA and ELA were complementary in targeting hepatic molecular mechanisms facilitating further attenuation of steatohepatitis and fibrogenesis in AMLN *ob/ob*-NASH mice. These findings provide preclinical proof-of-concept for combined FXR and PPAR-α/δ agonist-based therapies in NASH.

## Supplementary information

Supplementary information

## Data Availability

All data generated or analyzed during this study are included in this published article (and its Supplementary Information files).
