# Supplementary material for: Combined obeticholic acid and elafibranor treatment promotes additive liver histological improvements in a diet-induced ob/ob mouse model of biopsy-confirmed NASH
Source: Sci Rep. 2019 Jun 21;9:9046. doi: 10.1038/s41598-019-45178-z (PMC6588626; doi:10.1038/s41598-019-45178-z)
Supplement: Supplementary file 1 — Supplementary information [file 41598_2019_45178_MOESM1_ESM.pdf]

SUPPLEMENTARY INFORMATION

Combined obeticholic acid and elafibranor treatment promotes additive liver histological improvements in a diet-induced *ob/ob* mouse model of biopsy-confirmed NASH

Jonathan D Roth[1], Sanne S Veidal[2], Louise KD Fensholdt[2], Kristoffer TG Rigbolt[2], Romeo Papazyan[1], Jens Christian Nielsen[2], Michael Feigh[2], Niels Vrang[2], Mark Young[1], Jacob Jelsing[2], Luciano Adorini[1], Henrik H Hansen[2]*

[1]Intercept Pharmaceuticals, San Diego, CA; [2]Gubra, Hørsholm, Denmark

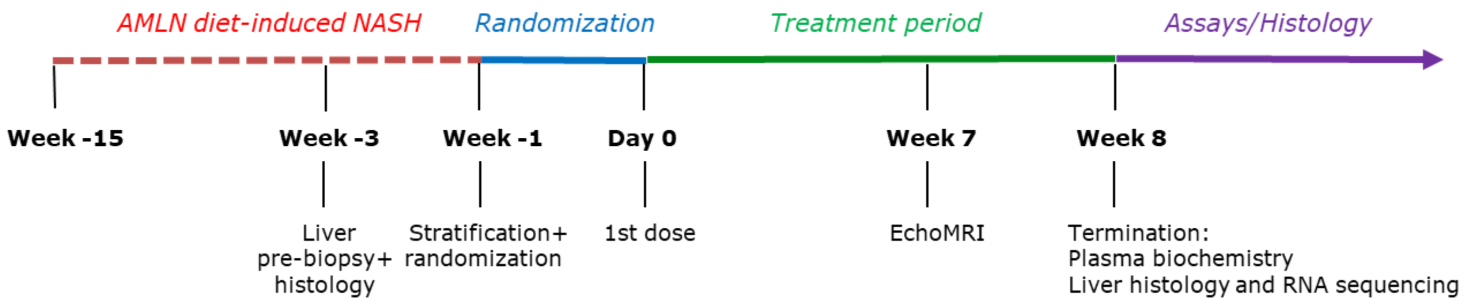

Supplementary Figure S1. Study outline. 6 weeks old *ob/ob* mice were fed the AMLN diet for 15 weeks prior to dosing start (day 0). A liver pre-biopsy was sampled in all AMLN *ob/ob*-NASH mice at three weeks prior to treatment start (week -3). Individual histopathology was evaluated for stratification of baseline NASH severity before dosing start. Animals were stratified and randomized to treatment groups based on mean fibrosis level (collagen 1a1 immunostaining, fractional area), then body weight (measured one week prior to treatment start, week -1). Body weight was monitored daily and 24h food intake was assessed once weekly during the treatment period. Body composition was analyzed in treatment week 7 by non-invasive EchoMRI scanning. Plasma biochemistry analysis was applied to terminal plasma samples from non-fasted mice. Liver histology was compared on all paired pre-biopsy and terminal samples, allowing for within-subject analysis of histological changes following 8 weeks of drug treatment. RNA sequencing was performed on terminal liver samples.

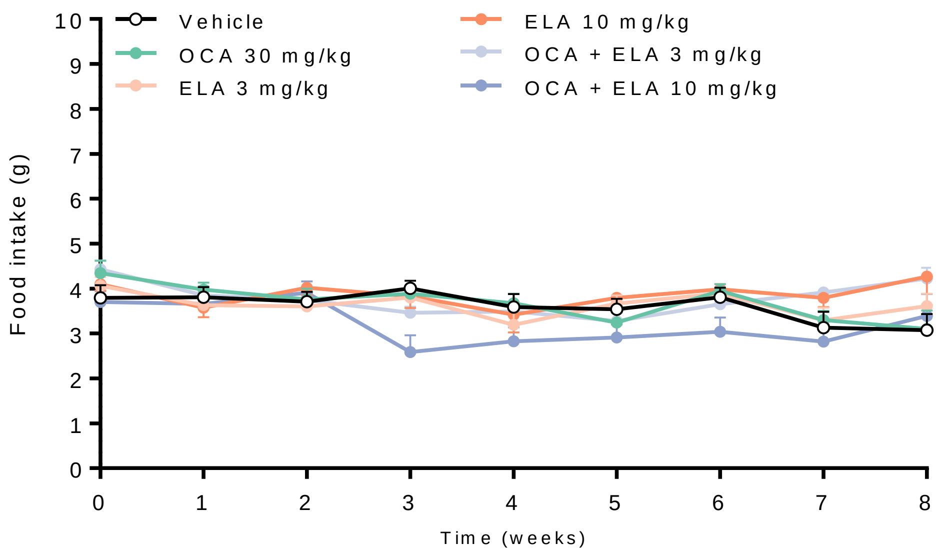

Supplementary Figure S2. Food intake in AMLN *ob/ob*-NASH mice treated with obeticholic acid (OCA), elafibranor (ELA) and combinations. Food intake was measured once weekly. Treatments had no effect on weekly food intake (overall $p>0.05$, two-way ANOVA with Tukey's multiple comparisons test).

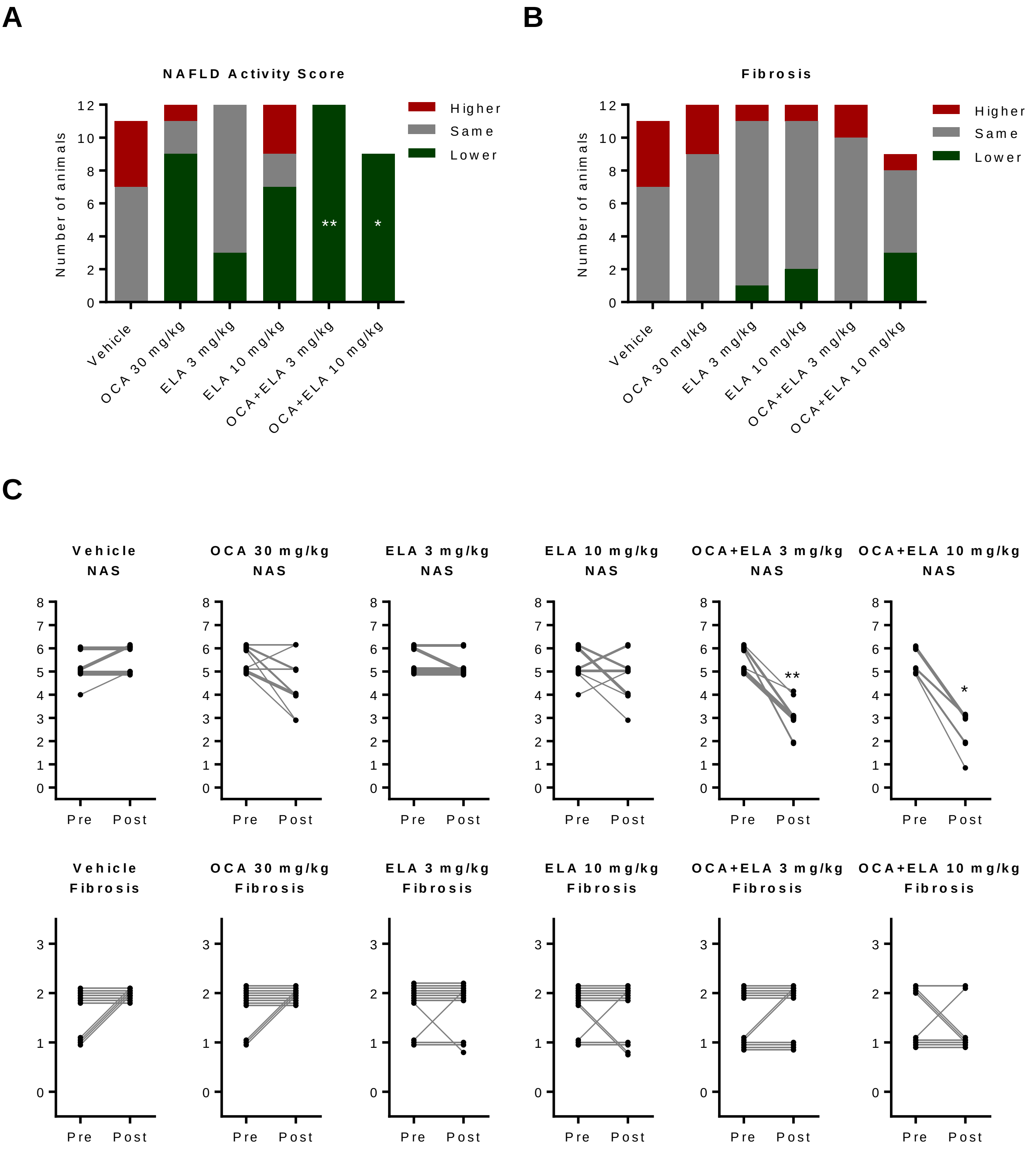

Supplementary Figure S3. Histomorphometric effects of obeticholic acid (OCA), elafibranor (ELA) and combinations in AMLN *ob/ob*-NASH mice. (A) Changes in NAFLD activity score, (B) Changes in fibrosis score, (C) Individual NAS and fibrosis scores. The NAFLD activity score (NAS) and fibrosis staging system was applied to paired liver pre-biopsy and terminal samples as outlined by Kleiner *et al.* (*Hepatology* 41: 1313-1321, 2005). Fisher's exact test with Bonferroni correction was used to test for within-subject changes in histology scores before and after treatment, compared to vehicle controls. *p<0.05, **p<0.01.

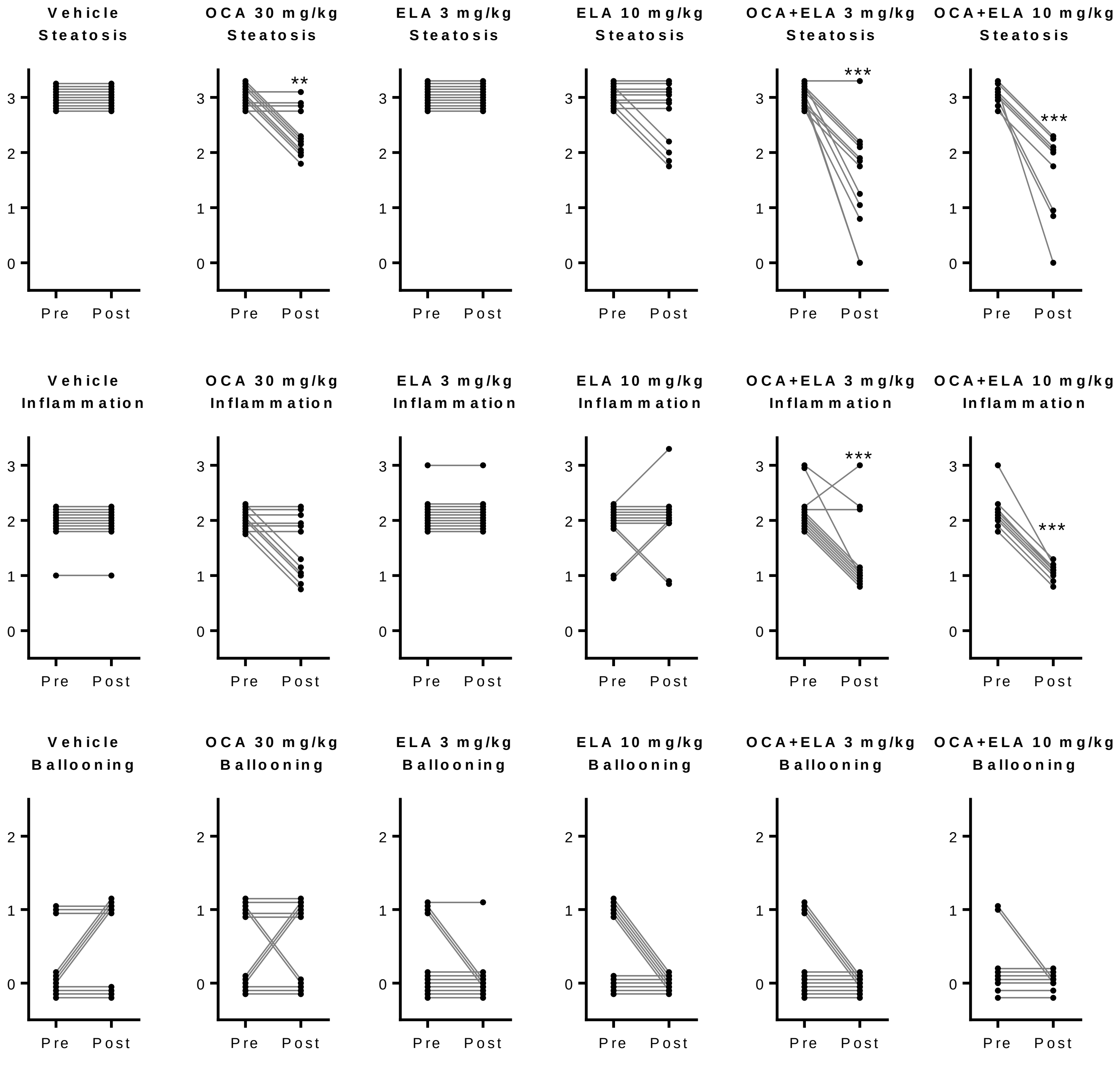

Supplementary Figure S4. Histomorphometric effects of obeticholic acid (OCA), elafibranor (ELA) and combinations in AMLN *ob/ob*-NASH mice. The NAFLD activity score (NAS) and fibrosis staging system was applied to paired liver pre-biopsy and terminal samples for scoring of steatosis, lobular inflammation and hepatocyte ballooning as outlined by Kleiner *et al.* (Hepatology 41: 1313-1321, 2005). Fisher's exact test was used to test for within-subject changes in histology scores before and after treatment, compared to vehicle controls. *p<0.05, **p<0.01, ***p<0.001.

**Accession key**	**Official symbol**	**Gene name**	**Gene set**	**Description**
ENSG00000110090	CPT1A	CPT1	Lipid metabolism	carnitine palmitoyltransferase 1A
ENSG00000169710	FASN	FASN	Lipid metabolism	fatty acid synthase
ENSG00000012504	NR1H4	FXR	Lipid metabolism	nuclear receptor subfamily 1 group H member 4
ENSG00000186281	GPAT2	GPAT2	Lipid metabolism	glycerol-3-phosphate acyltransferase 2, mitochondrial
ENSG00000158669	GPAT4	GPAT4	Lipid metabolism	glycerol-3-phosphate acyltransferase 4
ENSG00000113161	HMGCR	HMGCR	Lipid metabolism	3-hydroxy-3-methylglutaryl-CoA reductase
ENSG00000112972	HMGCS1	HMGCS1	Lipid metabolism	3-hydroxy-3-methylglutaryl-CoA synthase 1
ENSG00000134240	HMGCS2	HMGCS2	Lipid metabolism	3-hydroxy-3-methylglutaryl-CoA synthase 2
ENSG00000130164	LDLR	LDLR	Lipid metabolism	low density lipoprotein receptor
ENSG00000123384	LRP1	LRP1	Lipid metabolism	LDL receptor related protein 1
ENSG00000186951	PPARA	PPARA	Lipid metabolism	peroxisome proliferator activated receptor alpha
ENSG00000112033	PPARD	PPARD	Lipid metabolism	peroxisome proliferator activated receptor delta
ENSG00000073060	SCARB1	SCARB1	Lipid metabolism	scavenger receptor class B member 1
ENSG00000099194	SCD	SCD1	Lipid metabolism	stearoyl-CoA desaturase
ENSG00000104549	SQLE	SQLE	Lipid metabolism	squalene epoxidase
ENSG00000198911	SREBF2	SREBF2	Lipid metabolism	sterol regulatory element binding transcription factor 2
ENSG00000072310	SREBF1	SREBP1	Lipid metabolism	sterol regulatory element binding transcription factor 1
ENSG00000142208	AKT1	AKT1	Insulin signaling	AKT serine/threonine kinase 1
ENSG00000126767	ELK1	ELK1	Insulin signaling	ELK1, ETS transcription factor
ENSG00000150907	FOXO1	FOXO1	Insulin signaling	forkhead box O1
ENSG00000131482	G6PC	G6PASE	Insulin signaling	glucose-6-phosphatase catalytic subunit
ENSG00000229894	GK3P	GK	Insulin signaling	glycerol kinase 3 pseudogene
ENSG00000181856	SLC2A4	GLUT4	Insulin signaling	solute carrier family 2 member 4
ENSG00000177885	GRB2	GRB2	Insulin signaling	growth factor receptor bound protein 2
ENSG00000111713	GYS2	GYS2	Insulin signaling	glycogen synthase 2
ENSG00000171105	INSR	INSR	Insulin signaling	insulin receptor
ENSG00000169047	IRS1	IRS1	Insulin signaling	insulin receptor substrate 1
ENSG00000185950	IRS2	IRS2	Insulin signaling	insulin receptor substrate 2
ENSG00000100030	MAPK1	MAPK1	Insulin signaling	mitogen-activated protein kinase 1
ENSG00000102882	MAPK3	MAPK3	Insulin signaling	mitogen-activated protein kinase 3
ENSG00000169032	MAP2K1	MEK1	Insulin signaling	mitogen-activated protein kinase kinase 1
ENSG00000126934	MAP2K2	MEK2	Insulin signaling	mitogen-activated protein kinase kinase 2
ENSG00000124253	PCK1	PEPCK	Insulin signaling	phosphoenolpyruvate carboxykinase 1
ENSG00000163558	PRKCI	PKC	Insulin signaling	protein kinase C iota
ENSG00000067606	PRKCZ	PKC	Insulin signaling	protein kinase C zeta
ENSG00000100504	PYGL	PYG	Insulin signaling	phosphorylase, glycogen, liver
ENSG00000118137	APOA1	APOAI	FXR signaling	apolipoprotein A1
ENSG00000110243	APOA5	APOAV	FXR signaling	apolipoprotein A5
ENSG00000234906	APOC2	APOCII	FXR signaling	apolipoprotein C2
ENSG00000110245	APOC3	APOCIII	FXR signaling	apolipoprotein C3
ENSG00000083807	SLC27A5	BACS	FXR signaling	solute carrier family 27 member 5
ENSG00000167910	CYP7A1	CYP7A1	FXR signaling	cytochrome P450 family 7 subfamily A member 1
ENSG00000180432	CYP8B1	CYP8B1	FXR signaling	cytochrome P450 family 8 subfamily B member 1
ENSG00000005471	ABCB4	MDR3	FXR signaling	ATP binding cassette subfamily B member 4
ENSG00000137204	SLC22A7	OAT2	FXR signaling	solute carrier family 22 member 7
ENSG00000186198	SLC51B	OSTB	FXR signaling	solute carrier family 51 beta subunit
ENSG00000131910	NR0B2	SHP	FXR signaling	nuclear receptor subfamily 0 group B member 2
ENSG00000109181	UGT2B10	UGT2B4	FXR signaling	UDP glucuronosyltransferase family 2 member B10
ENSG00000147852	VLDLR	VLDLR	FXR signaling	very low density lipoprotein receptor
ENSG00000197442	MAP3K5	ASK1	Inflammation signaling	mitogen-activated protein kinase kinase kinase 5

ENSG00000170345	FOS	FOS	Inflammation signaling	Fos proto-oncogene, AP-1 transcription factor subunit
ENSG00000213341	CHUK	IKK	Inflammation signaling	conserved helix-loop-helix ubiquitous kinase
ENSG00000107643	MAPK8	JNK	Inflammation signaling	mitogen-activated protein kinase 8
ENSG00000177606	JUN	JUN	Inflammation signaling	Jun proto-oncogene, AP-1 transcription factor subunit
ENSG00000109320	NFKB1	NF-KB	Inflammation signaling	nuclear factor kappa B subunit 1
ENSG00000185386	MAPK11	P38	Inflammation signaling	mitogen-activated protein kinase 11
ENSG00000175387	SMAD2	SMAD2	Inflammation signaling	SMAD family member 2
ENSG00000166949	SMAD3	SMAD3	Inflammation signaling	SMAD family member 3
ENSG00000141646	SMAD4	SMAD4	Inflammation signaling	SMAD family member 4
ENSG00000105329	TGFB1	TGFB	Inflammation signaling	transforming growth factor beta 1
ENSG00000106799	TGFBR1	TGFBR	Inflammation signaling	transforming growth factor beta receptor 1
ENSG00000136869	TLR4	TLR4	Inflammation signaling	toll like receptor 4
ENSG00000232810	TNF	TNFA	Inflammation signaling	tumor necrosis factor
ENSG00000067182	TNFRSF1A	TNFR	Inflammation signaling	TNF receptor superfamily member 1A
ENSG00000127191	TRAF2	TRAF2	Inflammation signaling	TNF receptor associated factor 2
ENSG00000175104	TRAF6	TRAF6	Inflammation signaling	TNF receptor associated factor 6
ENSG00000163823	CCR1	CCR1	Monocyte recruitment	C-C motif chemokine receptor 1
ENSG00000121807	CCR2	CCR2	Monocyte recruitment	C-C motif chemokine receptor 2
ENSG00000170458	CD14	CD14	Monocyte recruitment	CD14 molecule
ENSG00000177575	CD163	CD163	Monocyte recruitment	CD163 molecule
ENSG00000129226	CD68	CD68	Monocyte recruitment	CD68 molecule
ENSG00000174837	ADGRE1	F4/80	Monocyte recruitment	adhesion G protein-coupled receptor E1
ENSG00000136634	IL10	IL-10	Monocyte recruitment	interleukin 10
ENSG00000115008	IL1A	IL-1A	Monocyte recruitment	interleukin 1 alpha
ENSG00000125538	IL1B	IL-1B	Monocyte recruitment	interleukin 1 beta
ENSG00000131981	LGALS3	MAC-2	Monocyte recruitment	galectin 3
ENSG00000108691	CCL2	MCP-1	Monocyte recruitment	C-C motif chemokine ligand 2
ENSG00000271503	CCL5	RANTES	Monocyte recruitment	C-C motif chemokine ligand 5
ENSG00000163568	AIM2	AIM2	Hepatocellular cell death	absent in melanoma 2
ENSG00000137752	CASP1	CASP1	Hepatocellular cell death	caspase 1
ENSG00000164305	CASP3	CASP3	Hepatocellular cell death	caspase 3
ENSG00000138794	CASP6	CASP6	Hepatocellular cell death	caspase 6
ENSG00000138794	CASP6	CASP6	Hepatocellular cell death	caspase 6
ENSG00000165806	CASP7	CASP7	Hepatocellular cell death	caspase 7
ENSG00000064012	CASP8	CASP8	Hepatocellular cell death	caspase 8
ENSG00000150782	IL18	IL18	Hepatocellular cell death	interleukin 18
ENSG00000091106	NLRC4	IPAF	Hepatocellular cell death	NLR family CARD domain containing 4
ENSG00000091592	NLRP1	NLRP1B	Hepatocellular cell death	NLR family pyrin domain containing 1
ENSG00000162711	NLRP3	NLRP3	Hepatocellular cell death	NLR family pyrin domain containing 3
ENSG00000137275	RIPK1	RIP1	Hepatocellular cell death	receptor interacting serine/threonine kinase 1
ENSG00000129465	RIPK3	RIP3	Hepatocellular cell death	receptor interacting serine/threonine kinase 3
ENSG00000107796	ACTA2	A-SMA	Stellate cell activation	actin, alpha 2, smooth muscle, aorta
ENSG00000076706	MCAM	CD146	Stellate cell activation	melanoma cell adhesion molecule
ENSG00000108821	COL1A1	COL1A1	Stellate cell activation	collagen type I alpha 1 chain
ENSG00000164692	COL1A2	COL1A2	Stellate cell activation	collagen type I alpha 2 chain
ENSG00000168542	COL3A1	COL3A1	Stellate cell activation	collagen type III alpha 1 chain
ENSG00000130635	COL5A1	COL5A1	Stellate cell activation	collagen type V alpha 1 chain
ENSG00000204262	COL5A2	COL5A2	Stellate cell activation	collagen type V alpha 2 chain
ENSG00000080573	COL5A3	COL5A3	Stellate cell activation	collagen type V alpha 3 chain
ENSG00000142156	COL6A1	COL6A1	Stellate cell activation	collagen type VI alpha 1 chain
ENSG00000142173	COL6A2	COL6A2	Stellate cell activation	collagen type VI alpha 2 chain
ENSG00000163359	COL6A3	COL6A3	Stellate cell activation	collagen type VI alpha 3 chain

ENSG00000138798	EGF	EGF	Stellate cell activation	epidermal growth factor
ENSG00000131095	GFAP	GFAP	Stellate cell activation	glial fibrillary acidic protein
ENSG00000137745	MMP13	MMP13	Stellate cell activation	matrix metallopeptidase 13
ENSG00000087245	MMP2	MMP2	Stellate cell activation	matrix metallopeptidase 2
ENSG00000100985	MMP9	MMP9	Stellate cell activation	matrix metallopeptidase 9
ENSG00000197461	PDGFA	PDGF	Stellate cell activation	platelet derived growth factor subunit A
ENSG00000102265	TIMP1	TIMP1	Stellate cell activation	TIMP metallopeptidase inhibitor 1
ENSG00000035862	TIMP2	TIMP2	Stellate cell activation	TIMP metallopeptidase inhibitor 2
ENSG00000100234	TIMP3	TIMP3	Stellate cell activation	TIMP metallopeptidase inhibitor 3
ENSG00000157150	TIMP4	TIMP4	Stellate cell activation	TIMP metallopeptidase inhibitor 4

Supplementary Table S1. Candidate genes associated with NAFLD and fibrosis.
